# Epithelial ingrowth in descemet membrane endothelial keratoplasty associated with vitreous loss

**DOI:** 10.1186/s12886-024-03370-4

**Published:** 2024-03-26

**Authors:** Anny M.S. Cheng, Anup Kubal, Shailesh K. Gupta, Anil S. Vedula, David T.Y. Yang, Aarup A. Kubal

**Affiliations:** 1 Department of Ophthalmology, Broward Health, Fort Lauderdale, FL USA; 2 Specialty Retina Center, Coral Springs, FL USA; 3 Department of Ophthalmology, Florida International University, Herbert Wertheim College of Medicine, Miami, FL USA; 4 Your Eye Specialists, 1776 N. Pine Island Rd., Suite 214, 33322 Weston, Plantation, FL USA; 5 University of California, Davis College of Biological Science, Sacramento, CA USA

**Keywords:** Descemet membrane endothelial keratoplasty, Epithelial ingrowth, Vitreous loss, YAG laser

## Abstract

**Background:**

Epithelial ingrowth is a rare but potentially sight-threatening complication caused by the invasion of corneal or conjunctival epithelial cells into the eye during ocular surgeries. DMEK is emerging as a widely used surgery for endothelial keratoplasty with its improved safety profile. We describe a case of epithelial ingrowth in the graft-host interface after uneventful DMEK associated with vitreous prolapse in the anterior chamber.

**Case presentation:**

An 81-year-old female with Fuchs endothelial dystrophy underwent DMEK for corneal decompensation following cataract surgery. During the DMEK procedure, vitreous prolapse was observed around the intraocular lens (IOL). Her early postoperative course was unremarkable, but a dense paracentral interface opacity was observed during the 3-month follow-up. The area of epithelial ingrowth was imaged with optical coherence tomography (OCT) as a uniform nodule with a discrete increase in interface hyperreflectivity. A low-energy YAG laser was applied to remove the opacity. She maintained good vision and clear cornea without reoccurrence after treatment.

**Conclusions:**

We propose that, in addition to the introduction of epithelial cells during surgery, vitreous retention in the anterior chamber may be a risk factor by providing a scaffold that potentially aggravates epithelial ingrowth in DMEK. Our case demonstrated that early YAG intervention may disrupt interface epithelial cell growth, and the transmitted laser energy may fragment the scaffold vitreous noninvasively.

## Introduction

Epithelial ingrowth is a rare complication of ocular surgery, with clinical manifestations ranging from asymptomatic to severe visual impairment and corneal melt. In the past, epithelial ingrowth was described after trauma, cataract surgery, penetrating keratoplasty (PKP), pterygium excision, glaucoma surgeries, and procedures in the anterior chamber, like aspiration or iris cyst excision.(reviewed in [1] ) Recently, epithelial ingrowth with the migration and growth of corneal or conjunctival epithelial cells into a lamellar interface after laser in situ keratomileusis (LASIK) or Descemet’s Stripping Automated Endothelial Keratoplasty (DSAEK) has been documented [2]. Descemet membrane endothelial keratoplasty (DMEK) is becoming the preferred endothelial keratoplasty surgical technique as it provides fast recovery, better visual outcomes, and fewer rejection rates [3–5]. Despite the demonstrated effectiveness and a superior safety profile of DMEK, a few cases of DMEK associated epithelial ingrowth have been identified [6–9].

The donor-cornea preparation may account for the donor epithelium origin of the invaded cells in DSAEK [2] or DMEK [8]. There is a reported case of dragging of recipient epithelial cells through peripheral incisions [9]. Many reported cases of DSAEK epithelial ingrowth involve graft detachment or dislocation with exposure of denuded endothelium and may facilitate the migration of donor epithelial cells [2]. However, the mechanism of how cell implantation in DMEK occurs remains unknown. In a 30-year clinicopathology review of 106 surgical eyes with epithelial ingrowth, 5.5% of the eyes had vitreous loss. Of these, 76 eyes (72%) and 15 eyes (15%) underwent intracapsular (ICCE) and extracapsular cataract extraction (ECCE), respectively. While surgical wound fistula and stromal vascularization have been hypothesized to facilitate epithelial downgrowth, the impact of vitreous loss remains unknown [1]. To our knowledge, this is one of the first reports of a patient with DMEK with vitreous loss developing epithelial ingrowth. Given that vitreous prolapse is a possible surgical complication of cataract surgery and DMEK is being advocated for more widespread use in patients, it was paramount to publish our experience when undergoing this therapy.

## Case report

An 81-year-old woman presented with left eye blurred vision and persistent corneal edema despite Sodium Chloride 5% (Bausch & Lomb Americas Inc., NJ, USA) treatment for 7 months following cataract surgery 1 year prior. Her best corrected visual acuity (BCVA) was 20/30 in the right eye and 20/100 in the left, and her intraocular pressure (IOP) measured with applanation tonometry was 16 mm Hg bilaterally. The anterior segment examination of the left eye revealed a well-centered intraocular lens with corneal stromal edema, confluent central guttata, and endothelial folds. The pachymetry measurement of the edematous central corneal thickness was 762 μm. The examination of the right eye was unremarkable, but endothelial cell counts by specular microscopy was 1574/mm^2^ with increased polymegathism and pleomorphism, whereas it was not possible to obtain measurements in the left eye. She therefore underwent DMEK in her left eye for Fuchs endothelial dystrophy. The patient’s cornea was first stripped of the 8-mm host Descemet membrane (DM), followed by an inferior peripheral iridotomy (PI) before graft insertion. Upon removal of viscoelastic, vitreous prolapse was observed around the intraocular lens (IOL), indicating that the patient had previously undergone a complicated cataract procedure. The stripped donor tissue was preloaded before being injected into the eye. In the process of shallowing the anterior chamber to unfold the graft, additional vitreous prolapsed. The vitreous was removed using a Weck-cell vitrectomy. Once the graft was unfolded and appropriately centered, 20% sulfur hexafluoride gas was placed under the donor graft for tamponade, and a final 80% fill was left at the end of surgery, along with a patent inferior PI. On the first postoperative day, the left eye of the patient demonstrated a BCVA of 20/800 and an elevated IOP of 39 mm Hg, but no pupillary block. Patient was started on brinzolamide and brimonidine tartrate ( Simbrinza, ALCON, INC., Texas, USA) three times per day, and her IOP decreased to 14 mm Hg the next day, with a BCVA of 20/125.

**Fig. 1:**
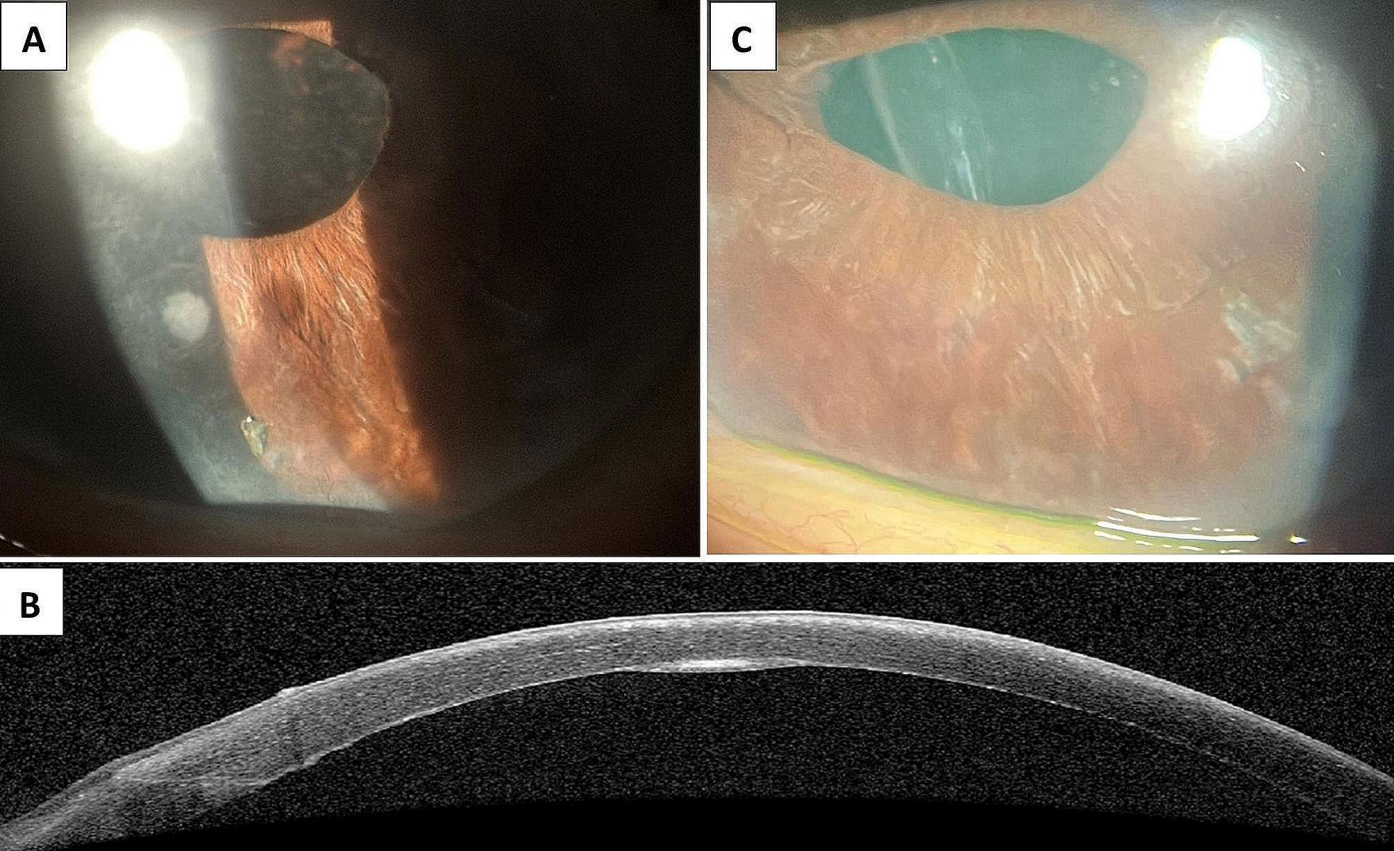
Image of Epithelial Ingrowth in Descemet Membrane Endothelial Keratoplasty Associated with Vitreous Loss A 1 mm dense paracentral interface opacity located at 7 o’clock with no anterior chamber inflammation during the 3-month follow-up after DMEK (**A**). Epithelial ingrowth imaged with optical coherence tomography (OCT) as a uniform nodule with a discrete increase in interface hyperreflectivity (**B**). The cornea remained clear without reoccurrence after YAG laser treatment (**C**)

Her early postoperative course was unremarkable. Anterior segment optical coherence tomography (OCT) imaging revealed that the graft was firmly attached. One month following DMEK surgery, specular microscopy revealed an endothelial cell density of 2,653/mm^2^. At the 2-month follow-up visit, she was on prednisolone acetate 1% three times daily; her BCVA improved to 20/40 with an IOP of 16 mmHg, and there was no evidence of intraocular inflammation. Donor corneal rim cultures were negative. At the 3-month follow-up, her vision remained unchanged, but an unexpected 1 mm dense paracentral interface opacity was observed at 7 o’clock (Fig. 1A). OCT showed the nodule had a uniform shape and a discrete increase in interface hyperreflectivity, which suggests the presence of epithelial ingrowth (Fig. 1B). The eye was first treated medically with 1% prednisolone acetate four times a day, given that the epithelial ingrowth lesion was small, and the patient did not have a significant vision disturbance. At the 4.5-month follow-up visit (1.5 months after epithelial ingrowth), her left eye vision was 20/30, with the same size of interface epithelial lesion. The patient was counseled about possible epithelial advancement and decreased vision. After a discussion of the options of observation versus intervention, consent was obtained to proceed with laser treatment. A YAG laser (Carl Zeiss Meditec AG, Germany) of 39 pulses at 0.9 mJ was applied to remove the opacity. The offset control on the YAG laser was set to zero, and the laser was aimed at the graft-host interface. A slightly visible bubble appeared immediately with shots, and the interface opacity was instantly and clearly visible removed upon laser application. By targeting the central island of epithelial cells and the lesion edge with low energy pulses, laser energy was prevented from reaching adjacent endothelial cells to avoid collateral damage. Prednisolone acetate was tapered accordingly. At the 1-month follow-up visit after laser treatment, the dense area of opacification had completely resolved with a clear interface. Four months post-laser treatment, the cornea was clear without reoccurrence (Fig. 1C). Her BCVA was 20/30, with a corneal thickness of 510 mm.

## Discussion

Epithelial ingrowth with the introduction of corneal or conjunctival epithelial cells into the eye is a very uncommon but potentially catastrophic intraocular surgical complication. Graft-host interface epithelial ingrowth following LASIK and DSAEK has been documented, and more recently, after a few cases of DMEK [6–9]. In previous published DMEK cases, the cause of the ingrowth was unknown and was proposed to be either from the donor and delivered during tissue preparation or originating from the recipient and dragged through peripheral incisions [7, 9]. In our case, epithelial ingrowth within the graft host interface occurred after uneventful DMEK associated with vitreous loss in the anterior chamber. We propose that, in addition to the introduction of epithelial cells during surgery, prolapsed vitreous may function as a scaffold to facilitate interface membrane ingrowth. This notion is supported by a clinical observation in anterior proliferative vitreoretinopathy that the residual vitreous can serve as a scaffold for membranes containing proliferating cells or extracellular matrix deposition. The incidence of epithelial ingrowth ranged from 0.076 to 0.12% in an extensive review of 444,496 cataract surgeries, including ICCE and ECCE [1]. Vitreous loss was widespread in these individuals, even though it was unclear if it was a predisposing factor in the development of epithelial ingrowth. Similarly, a case report of repeated failed DSAEK complicated with vitreous prolapse within the surgical wound revealed conjunctival epithelial ingrowth [10]. This further supports the notion that vitreous retention can be a risk factor for epithelial ingrowth by providing a platform for epithelial migration adjacent to an incision.

Clinically, epithelial ingrowth manifests as a homogenous white mass composed of clusters containing amorphous materials with few cellular elements indicating a decrease in proliferation at the late stage. This is consistent with the observation that OCT demonstrates hyperreflective masses in our case and/or hyporeflective clefts, which may represent various layers of epithelium trapped at the interface between the flap and stromal bed. While epithelial ingrowth can be a contributing factor in interface opacity seen following DMEK, other potential causes include infection and inflammation. Initial intraocular inflammation in our patient following DMEK was minimal, and the eye was quiet without inflammation when interface opacity developed. In addition, our donor corneal margin cultures were negative, which, in conjunction with the OCT finding, makes an infectious etiology unlikely.

Treatment options for epithelial ingrowth may vary based on the progression and visual impact of epithelial ingrowth. Several previous studies have suggested observation, antimetabolite therapy, surgical resection with adjunctive cryotherapy, PKP, or repeat keratoplasty [2]. In cases of severe epithelial ingrowth interface abnormalities, irrigation and aspiration of the residual epithelial cells and repeat keratoplasty may achieve a favorable outcome. In the advanced stages of extrainterface extension with stromal opacity or graft failure, PKP will ultimately be needed to remove epithelial cells completely. However, in addition to the potential risk of dispersing epithelial cells in the anterior chamber, repeated keratoplasty or PKP involves a surgically invasive risk. Recent research indicates that YAG laser treatment for epithelial ingrowth after DMEK is a less invasive option for patients with a clear graft and good vision [7]. Although observation may be an initial option when there is no sign of progression, we chose to proceed with YAG laser therapy in this case. We believe vitreous retention potentially aggravates epithelial ingrowth despite a few studies reported that the progression of epithelial ingrowth may be halted by the epithelial–endothelial contact inhibition of movement [11]. In addition, when YAG laser is used to disrupt interface epithelial ingrowth following endothelial keratoplasty, the transmitted laser energy may fragment or disorganize the scaffold vitreous noninvasively. Our case demonstrated that early YAG laser intervention may successfully treat interface epithelial ingrowth in DMEK with residual vitreous. Further study is warranted to identify the role of epithelial cells in epithelial ingrowth in order to clarify the associated pathophysiology and optimize treatment.

## Data Availability

All data generated or analysed during this study are included in this article.

## Abbreviations

PKP: penetrating keratoplasty
LASIK: Laser-Assisted In Situ Keratomileusis
DSAEK: Descemet’s Stripping Automated Endothelial Keratoplasty
DMEK: Descemet membrane endothelial keratoplasty
BCVA: best corrected visual acuity
IOP: intraocular pressure
ICCE: intracapsular cataract extraction
ECCE: extracapsular cataract extraction
DM: Descemet membrane
PI: peripheral iridotomy
IOL: intraocular lens
OCT: optical coherence tomography
